# Genomic and functional characterization of *Pseudosulfitobacter pseudonitzschiae* BPC-C4-2: a growth-promoting symbiont in Antarctic *Ulva* communities

**DOI:** 10.1186/s12864-026-12626-w

**Published:** 2026-02-07

**Authors:** Tia Wünschmann, Fatemeh Ghaderiardakani, Timo Homeier-Bachmann, Maria Liliana Quartino, Thomas Wichard, Anne Busch

**Affiliations:** 1 Theoretical Microbial Ecology, Friedrich Schiller University, Jena, Germany; 2 Cluster of Excellence Balance of the Microverse, Friedrich Schiller University Jena, Jena, Germany; 3 Institute for Inorganic and Analytical Chemistry, Friedrich Schiller University, Jena, Germany; 4 Friedrich-Loeffler-Institute, Institute of Epidemiology, Greifswald, Insel Riems Germany; 5 Department of Coastal Biology, Argentinean Antarctic Institute, Buenos Aires, Argentina

**Keywords:** *Pseudosulfitobacter pseudonitzschiae*, Phylogenetic clustering, Genomic analyses, *Ulva* sp., *Roseobacteraceae*, Antarctic ecosystems, *Maribacter*

## Abstract

**Background:**

*Pseudosulfitobacter pseudonitzschiae* is a species within the genus *Pseudosulfitobacter*, which belongs to the *Roseobacteraceae*. This family is closely associated with algae and is essential to marine ecosystems, particularly through interactions with phytoplankton. Notably, this bacterium can produce bioactive compounds that influence microbial dynamics and algal growth in marine environments. In addition to essential nutritional factors, the marine green macroalgal genus *Ulva* (Chlorophyta) relies on a combination of regulatory morphogenetic compounds produced by its associated epiphytic bacteria to achieve proper morphogenesis. Since *P. pseudonitzschiae* is rarely described and phylogenetic clustering within this genus is challenging, we conducted phylogenetic and genomic analyses to better resolve its taxonomic position and to explore its functional potential in the Antarctic environment.

**Results:**

*P. pseudonitzschiae* BPC-C4-2 was isolated and integrated into a tripartite model system alongside *Maribacter* sp. BPC-D8 (CP128187.1) and *Ulva* sp. UPC-109 (PP091299.1). This biosystem was designed to study the mechanisms of cold-water adaptation involved in the morphogenetic development of the genus *Ulva*.

The hybrid genome assembly of *P. pseudonitzschiae* BPC-C4-2 consisted of seven contigs totaling 5,450,390 bp, with a GC content of 59.0%. Genome annotation identified 5,380 coding sequences (CDSs), 6 rRNA genes, 85 tRNA genes, and 1 tmRNA. The relatively large number of coding sequences and RNA genes observed may reflect an expanded genetic toolkit that enables metabolic flexibility and stress tolerance, potentially supporting adaptation to the extreme conditions of Antarctic and cold-water environments.

Given the taxonomic complexity within the bacterial family, both 16S rRNA gene sequencing and average nucleotide identity (ANI) analyses were conducted. Using affinity propagation clustering, these analyses enabled a more robust phylogenetic placement of *P. pseudonitzschiae* within this challenging group, providing deeper ecological and evolutionary insights.

In this study, we searched for gene clusters associated with metabolic adaptations. Functionally, the genome harbors a modularly organized sox gene cluster involved in the sarcosine oxidation pathway and dimethylsulfoniopropionate (DMSP) degradation. Additional pathways involved in osmolyte metabolism and methylation were also identified and found to be phylogenetically distinct from closely related species.

**Conclusions:**

Our findings provide the basis for a cold-water bioassay system to study the morphogenesis of *Ulva* collected from polar regions, in association with *Maribacter* sp. BPC-D8 and *P. pseudonitzschiae* BPC-C4-2. Genomic analyses of *P. pseudonitzschiae* BPC-C4-2 provide insights into its revised phylogeny based on affinity propagation clustering, along with a detailed analysis of genes involved in key metabolic pathways.

**Supplementary Information:**

The online version contains supplementary material available at 10.1186/s12864-026-12626-w.

## Background

Studying the genomes of Antarctic bacteria associated with algae can reveal unique adaptations to extreme environments that deepen our understanding of their roles in marine ecosystems and their influence on climate. Bacteria associated with macroalgae are known to induce algal development by releasing essential algal growth- and morphogenesis-promoting factors (AGMPFs) [1]. However, temperate-adapted AGMPF-producing strains often fail to grow at low temperatures (< 5 °C) or do not synthesize key morphogenetic compounds, such as thallusin [2]. To investigate cold-water adaptation in the morphogenetic development of *Ulva*, we established a tripartite system composed of the Antarctic *Pseudosulfitobacter pseudonitzschiae* BPC-C4-2 and *Maribacter* sp. BPC-D8 (CP128187.1), using the Mediterranean green macroalga *U. mutabilis* as a model [3]. To complete the genetic characterization of this system, we analyzed the previously uncharacterized genome of *P. pseudonitzschiae* BPC-C4-2. In the absence of bacteria that release algal growth- and morphogenesis-promoting factors (AGMPFs), the green macroalga and model organism *Ulva compressa* (cultivar *U. mutabilis*) develops into an undifferentiated, callus-like morphotype [4]. Two essential symbiotic bacteria, *Maribacter* sp. MS6 and *Roseovarius* sp. MS2, restore normal growth and development, thus forming the tripartite community of temperate *U. mutabilis*–*Maribacter* sp. MS6–*Roseovarius* sp. MS2. Within this partnership, *Maribacter* sp. serves a pivotal role in promoting rhizoid formation and cell wall development in *Ulva*, a process triggered by the AGMPF (−)-thallusin [4]. The AGMPF produced by *Roseovarius* sp. exhibits cytokinin-like activity, similar to the action of plant hormones [5]. Notably, this reductionistic model system can be studied under standardized conditions (e.g., in a changing environment) [6].

While cold-adapted thallusin-producing strains have been well characterized [2], the third partner in the tripartite system, which phenocopies the temperate strain *Roseovarius* sp. MS2 [2], has remained uncharacterized until now. Therefore, *Pseudosulfitobacter pseudonitzschiae* BPC-C4-2 was selected for genome sequencing because it emerged from a morphogenetic survey of Antarctic bacterial isolates as a consistent partner in the cold-adapted tripartite bioassay, phenocopying the functional role of the temperate *Roseovarius* strain by promoting *Ulva* cell division and growth. In addition, its genome had not previously been characterized in the context of algal morphogenesis under polar conditions.

This strain was isolated in February 2020 from the surface of *Ulva* sp. UPC-109 obtained from Potter Cove, Isla 25 de Mayo/King George Island, Antarctica. It was collected along with many species of the *Roseobacteraceae*, which include the *Sulfitobacter* and *Roseobacter* lineages as significant subgroups within the class *Alphaproteobacteria*, according to the National Center for Biotechnology Information (NCBI) taxonomy and LPSN (https://lpsn.dsmz.de/species/pseudosulfitobacter-pseudonitzschiae). These lineages contain different environmental adaptations and possess distinct genomic, phylogenetic, and in silico-predicted phenotypic data [7–9].

Members of the *Roseobacter* lineage can account for up to 20% of coastal marine bacterial populations, ranking among the most abundant marine bacterial groups [10]. *Roseobacter* strains are widely distributed across diverse ocean habitats, including coastal waters and marine sediments [11] and offshore [12]. They are also present on algal surfaces, such as those of diatoms [8] and macroalgae [13]. Notably, they are considered opportunistic taxa and are more prevalent in nutrient-rich nearshore waters [14]. These taxa have large, flexible genomes that can take advantage of higher concentrations of nutrients and more varied resource types [12, 15]. *Sulfitobacteria* are capable of oxidizing sulfite and thiosulfate, as well as degrading dimethylsulfoniopropionate (DMSP) and dimethylsulfoxoniumpropionate (DMSOP), which are organic sulfur-containing compounds that serve crucial roles in marine ecosystems and are climate-relevant [16].

Genomic analysis of the previously uncharacterized strain *P. pseudonitzschiae* BPC-C4-2 provides novel insights into its genomic structure, taxonomic classification, and the genes involved in the metabolism of osmolytes, such as DMSP and ectoine, which are crucial for bacteria–macroalgae interactions [17] and DNA methylation processes.

Using a hybrid genome assembly, we analyzed *P. pseudonitzschiae* BPC-C4-2 to study its phylogeny and genomics. Phylogenetic analysis was conducted using both 16S rRNA gene sequences [18] and average nucleotide identity (ANI) [19], with ANI serving as a reference-independent, alignment-free method, as previously described. Affinity propagation clustering (APC) revealed distinct clusters corresponding to previously described subgroups [20], with the advantage that APC can be applied independently and without annotation since it is mathematically derived, thereby reducing potential biases.

Given the organism’s cold-adapted environment near an *Ulva* host, particular attention was paid to genes involved in stress protection, nutrient acquisition, and osmolyte metabolism (e.g., sulfur and ectoine). The genetic content was analyzed for organic sulfur cycling metabolism and ectoine biosynthesis, functions that may support survival in Antarctic marine conditions. Both substance classes were previously identified as essential in bacteria–*Ulva* interactions [2, 3]. The sulfur oxidation Sox system has been described in many chemolithotrophic sulfur-oxidizing bacteria, such as *Paracoccus pantotrophus* and *Thiobacillus* species, in which a periplasmic Sox enzyme complex (soxXYZABCD) catalyzes the stepwise oxidation of reduced sulfur compounds to sulfate, thereby supporting energy conservation in sulfur-rich environments [21]. Ectoine biosynthesis gene clusters are commonly found in halophilic and cold-tolerant bacteria, such as *Halomonas* or *Marinobacter* species [22]. These clusters, which include genes such as *ectA*, *ectB*, and *ectC*, facilitate the production of ectoine [12, 23], an osmolyte synthesized under extreme environmental conditions to stabilize proteins and cellular structures [24]. Although ectoine was shown to be more actively produced at low temperatures (e.g., 2 °C), it is not directly biosynthesized by *Ulva* [1, 2].

Our findings offer deeper insights into the cold adaptation of the Antarctic strain *P. pseudonitzschiae* BPC-C4-2, thereby completing the characterization of the tripartite system underlying morphogenetic development in *Ulva* in cold-water environments.

## Methods

### Bacteria isolation, DNA isolation, and morphogenetic bioassay

*P. pseudonitzschiae* BPC‑C4‑2 and *Maribacter* sp. BPC‑D8 were sampled in February 2020 at Isla 25 de Mayo/King George Island (Potter Cove) from the green macroalga *Ulva* sp. UPC‑109, a strain closely related to *U. stenophylla* (based of tufa analysis) [2]. The bacteria were subsequently cultivated in marine broth (Roth, Germany) for 2 weeks at 2 °C. The isolate was stored at − 80 °C in marine broth and 15% glycerol. Genomic DNA was extracted using the PureLink™ Genomic DNA Mini Kit (Thermo Fisher Scientific) following the manufacturer’s protocol.

To assess the activity of potential morphogenesis-inducing bacteria, a morphogenetic bioassay was conducted using axenic cultures of *Ulva mutabilis* (strain FSU-UM5-1; recently reclassified in *U. compressa*) in 96 multi-well plates [14]. Axenic gametes of *U. mutabilis* were prepared [25] and counted by flow cytometry [26]. Thereafter, gametes were inoculated with the bacterial isolates in 200 µl of Ulva culture medium (UCM). After inoculation, the final optical density of the bacteria was OD_620_ = 1.0 × 10^−4^ with 20–50 gametes in 200 µl of UCM. We qualitatively investigated bacterial effects by specifically screening for a phenocopy of *Roseovarius* sp. MS2, carried out in a previous study [2], in order to identify a cold-adapted bacterial partner for subsequent genomic characterization. As positive controls, the AGMPF-releasing reference strains *Roseovarius* sp. (GenBank: EU359909) and *Maribacter* sp. (GenBank: EU359911) were inoculated individually and in combination with the axenic cultures [2, 5]. *U*. *mutabilis* was grown in UCM (50 ml) under standardized laboratory conditions, with a 17/7 h light/dark cycle and a light intensity of 40–80 mol photons m^2^ s^−1^ at 18 °C ± 2 °C [27]. All treatments and controls were performed in triplicate. The *Ulva* morphogenetic bioassay array relies on the saturating release of algal growth- and morphogenesis-promoting factors (AGMPFs) by associated bacterial strains, ensuring robust induction of algal developmental responses [14].

### Sequencing and base calling

Genomic DNA was quantified using a Qubit v.3.0 fluorometer (Invitrogen, USA) and a Qubit dsDNA HS kit (Invitrogen) according to the manufacturer’s protocol. A short-read sequencing library was prepared using a Nextera DNA Flex library preparation kit (Illumina, USA) and Nextera DNA CD indexes (Illumina) following the manufacturer’s protocol. A total of 1,826,456 paired-end reads (coverage × 147.3) were generated using an iSeq 100 instrument (Illumina) for paired-end sequencing (2 × 150 bp). A long-read sequencing library was prepared using a one-dimensional ligation sequencing kit SQK-LSK109 (Oxford Nanopore Technologies [ONT]) and a NEBNext Companion Module for ONT Ligation Sequencing kit (New England BioLabs, USA) following each manufacturer’s protocol. No size selection or shearing was applied. The library was loaded into a FLO-MIN106 flow cell (R9.4.1, ONT) on a MinION sequencer (ONT). Fast5 reads were then base-called using a MinKNOW v.19.10.1 (ONT) with Guppy v.3.3.3 in high-accuracy base-calling mode, generating 1,824,000 reads (coverage × 269.3) with an N50 of 1,919 bp. FastQC v.0.11.7 was employed to assess the sequence quality of both the Illumina and ONT libraries [28].

### Bioinformatics: Genome assembly and annotation

The quality of the nanopore reads was analyzed using FastQC (Galaxy version 0.74 + galaxy1) and Nanoplot (Galaxy version 1.43.0 + galaxy0) [29]. Genome assembly was performed using the Flye de novo assembler (Galaxy version 2.9.5 + galaxy0) for the nanopore reads [30]. The assembly was then polished using the medaka consensus pipeline (Galaxy version 1.7.2 + galaxy1) [31]. Illumina reads were mapped to the polished consensus sequence using BWA-MEM2 (Galaxy version 2.2.1 + galaxy1) [32]. Hybrid assembly was performed using pilon (Galaxy version 1.20.1) [33]. All steps were performed using the Galaxy platform [12].

For *Pseudosulfitobacter pseudonitzschiae* BPC-C4-2 (taxid:1,402,135), data are available from the NCBI under accession numbers PRJNA828511, SAMN47449418, and JBMGNR000000000.

### 16S rRNA gene and genome sequence collection

The 16S rRNA gene sequences of the type strains of validated species within the genus *Pseudosulfitobacter*, as well as those of closely related species, were downloaded from the NCBI GenBank database in August 2024 (https://www.ncbi.nlm.nih.gov; see accession numbers in Supplementary Table 1). To perform a comprehensive phylogenomic analysis of the genus *Pseudosulfitobacter*, 15 genome sequences of related type strains within the genera *Roseobacter*, *Sulfitobacter*, *Heliomarina*, and *Hyphomarina* were downloaded from the NCBI (https://www.ncbi.nlm.nih.gov) in August 2024. The genome sequence of *Hyphomonas* polymorpha was used as an outgroup in the phylogenomic analysis, as in previous studies [34].

### 16S rRNA gene-based and genome-based phylogenetic analyses

Multiple sequence alignment of the obtained 16S rRNA gene sequences was performed using the MAFFT alignment program [35] included in Geneious software (v20254.0. [14]. A phylogenetic tree was then constructed using the neighbor-joining method [36] and the Tamura-Nei genetic distance model [37] with the Geneious Tree Builder. Consensus tree options were set to the default settings. The tree-supported topologies were evaluated using bootstrap values from 100 replicates.

### Comparative genome analysis

The genomes and statistics of closely related *P. pseudonitzschiae* SMR1 and *Sulfitobacter pseudonitzschiae* H3 were obtained from the NCBI database. Average nucleotide identity was analyzed using the EZBiocloud ANI Calculator (https://www.ezbiocloud.net/tools/ani) [19]. To compare the occurrence of mobile elements here with those in other studies, PlasFlow (Galaxy v1.1.0 + galaxy0) [38] and PlasmidFinder (Galaxy Version 2.1.6 + galaxy1) [39] were used on the assembled genome of *Pseudosulfitobacter pseudonitzschiae* BPC-C4-2 strain. Affinity propagation clustering was used as previously described [20, 40, 41].

### Functional annotation and pathway analysis

The genome was annotated using Prokka (Galaxy version 1.14.6 + galaxy1) [42]. Annotation was performed under the "Bacteria" kingdom setting using the standard bacterial genetic code (Translation Table 11). Only contigs with a minimum of 200 base pairs were included in the analysis. The GFF3 output format was selected, and locus tags were automatically incremented by one for each annotated feature. Annotation was performed using a similarity e-value cutoff of 1e-6. Default Prokka databases were used for functional assignment, and no genus-specific BLAST database or trusted protein set was specified. Searches for rRNA genes (using Barrnap) and transfer RNA (tRNA) genes (using Aragorn) were enabled, while the search for non-coding RNAs via Rfam was disabled.

Annotated genome files obtained from the NCBI GenBank assembly database were manually reviewed to identify genes related to the sulfite oxidation and DMSP degradation pathways. Functional annotation of the bacterial genome was conducted using BlastKOALA (v3.1; https://www.kegg.jp/blastkoala/) [43] using the KEGG database (http://www.genome.jp/kegg/) [44]. Genes related to the sulfite oxidation and DMSP degradation pathways [21] were manually identified in the output within annotation files.

### Comparative genome alignment and structural variation analysis

Genome assembly was performed using Unicycler (Galaxy Version 0.5.1 + galaxy0) in hybrid assembly mode [45]. The bridging mode was set to normal, and contigs smaller than 200 bp were excluded from the assembly. Alignment was performed in Galaxy using Canu assembler (Galaxy Version 2.2 + galaxy0) with the default settings [46]. Alignment was also performed using the Mauve alignment tool included in Geneious software (v2025 4.0.) [47]. Both genomes were annotated using Prokka (Galaxy version 1.14.6 + galaxy1) with the same settings used for the Flye assembly [42].

## Results and discussion

### Establishing a standardized bioassay with axenic *Ulva* cultures to evaluate the morphogenetic development of bacteria isolated from polar regions

**Fig. 1:**
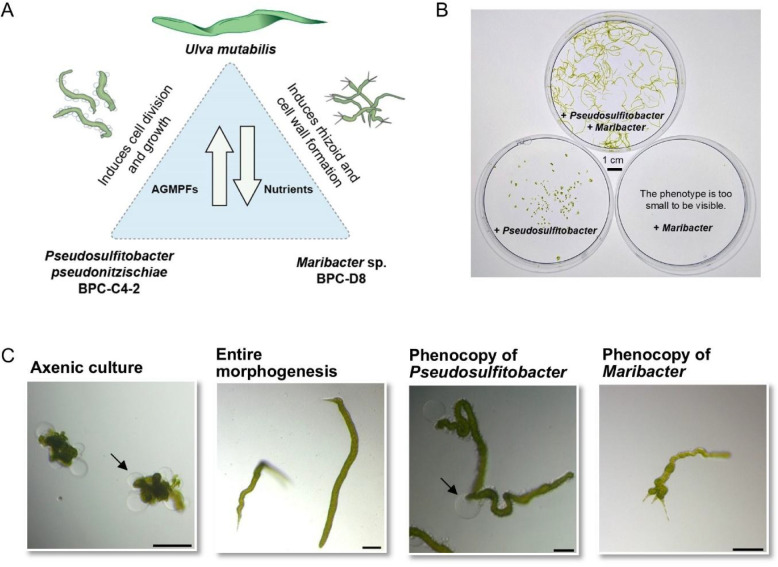
Model system *Ulva* **A** Representative morphotypes of the tripartite community consisting of *Ulva*, *P. pseudonitzschiae* BPC-C4-2 and *Maribacter* sp. BPC-D8. The illustration is adapted from Alsufyani et al. [4], with elements obtained from Wichard (2023) [48] (under the CC BY 4.0 license). **B** Bioassay using a purified gamete stock solution of *U. mutabilis*, tested in Petri dishes with and without the presence of *Maribacter* and *Pseudosulfitobacter*, showing 1-month-old cultures. The combination of both bacteria supports the full growth and morphogenesis of *Ulva* within the tripartite community. **C** Microscopic analysis of a 2–3-week-old culture following the inoculation of axenic *Ulva* gametes with specific bacteria. Axenic controls formed undifferentiated callus structures with characteristic cell wall protrusions (arrow). Although *Pseudosulfitobacter* induced cell division and growth, protrusions remained. *Maribacter* promoted rhizoid development and cell wall formation. Scale bar = 100 µm

Based on the results of previous studies [2, 14], we established a tripartite system comprising *P. pseudonitzschiae* BPC-C4-2, *Maribacter* sp. BPC-D8, and *U. mutabilis* to investigate cold-water-adapted bacteria in the morphogenetic development (Fig. 1A). The bioassay performed with axenic gametes of *U. mutabilis* (negative control) developed into a callus-like morphotype. In contrast, full growth and morphogenesis were observed in the tripartite community of *Ulva–Pseudosulfitobacter*–*Maribacter* at 18 °C (Fig. 1B). Although these bacteria originated from Antarctica, they could induce growth and morphogenesis in the temperate *U. mutabilis*. In contrast, temperate AGMPF-producing bacteria did not grow or release AGMPFs when shifted to polar temperatures, as previously reported [2]. *P. pseudonitzschiae* alone induced cell division, leading to wrinkled morphotypes (Fig. 1B) that lacked rhizoids and exhibited malformed cell walls (Fig. 1C). However, co-inoculation with *P. pseudonitzschiae* BPC-C4-2 and the thallusin-releasing *Maribacter* sp. BPC-D8 restored full development of the axenic gametes (Fig. 1C). Unlike the well-established temperate tripartite systems with *U. compressa* (cultivar *U. mutabilis*), *U. linza*, or *U. intestinalis *[48], the regulation of gametogenesis in the cold-adapted *Ulva* sp. UPC-109 requires further investigation, particularly to enable the induction of gametogenesis under controlled conditions and thus the production of gametes for establishing axenic cultures [5].

### Genomic analysis

Whole-genome sequences were generated with long reads (ONT) and short reads (Illumina). A short-read assembly was generated by leveraging the advantages of short reads, which have fewer errors, thereby improving base-level accuracy. The short-read assembly had 76 contigs and was 0.9% shorter than the hybrid assembly with 7 contigs (Supplementary Table 2). The hybrid assembly resulted in seven contigs, demonstrating superior assembly quality and structural accuracy. The GC content remained consistent across both assemblies (59.0%). The hybrid genome assembly was then compared to its closest phylogenetic neighbors (Table 1).

**Table 1:** Genomic comparison of *Pseudosulfitobacter pseudonitzschiae* BPC-C4-2 with closely related strains

	*Pseudosulfitobacter pseudonitzschiae* BPC-C4-2, JBMGNR000000000	*Pseudosulfitobacter pseudonitzschiae* SMR1, (CP022415-CP022422)	*Sulfitobacter pseudonitzschiae* H3, (NZ_JAMD00000000.1)
Contigs	7	8	-
Bases	5,450,390	5,121,602	4,945,295
CDS	5380	4938	4833
rRNA	6	6	6
tRNA	85	43	41
tmRNA	1	1	1
GC [%]	58.98	59.5	61.7
Circularisation	All contigs	All contigs	All contigs
ANI		99.79	84.3

BPC-C4-2 was compared with *P. pseudonitzschiae* SMR1 and *Sulfitobacter pseudonitzschiae* H3, highlighting differences in key genomic parameters

Genomes of *Pseudosulfitobacter pseudonitzschiae* BPC-C4-2 were compared with its closest neighbors, *Pseudosulfitobacter pseudonitzschiae* SMR1 CP022415-CP022422 and [49] *Sulfitobacter pseudonitzschiae* H3 NZ_JAMD00000000 [21, 49], across contigs, highlighting differences in base pairs, CDS, rRNA, tRNA, tmRNA, GC content, and ANI, revealing variations in genomic size, gene content, and evolutionary relationships (Table 1).

Base pair counts ranged from 4,945,295 to 5,450,390, while CDS counts ranged from 4,833 to 5,380. Notably, *P. pseudonitzschiae* BPC-C4-2 had the largest genome and the most gene content. Since tRNA is ubiquitous and abundant, its abundance is a critical parameter for gene synthesis and adaptability to adverse environments [50, 51]. While rRNA counts remained consistent at six across all contigs, tRNA numbers differed significantly, ranging from 38 to 85. This elevated tRNA content may enhance translational efficiency and adaptability, possibly providing a selective advantage under specific environmental or physiological conditions [52]. Furthermore, the GC content was very high, ranging from 59.0 to 63.0%, with the lowest GC content in *P. pseudonitzschiae* BPC-C4-2 suggesting possible cold-water adaptation. Moreover, ANI showed high similarity (99.7%) with *Pseudosulfitobacter pseudonitzschiae* SMR1 but lower similarity (84.3%) with *S. pseudonitzschiae* H3, reflecting a previously reported evolutionary divergence [34]. Notably, these differences highlight strain-specific adaptations and potential functional diversity.

### Analysis of mobile elements

We analyzed seven contigs for potential mobile elements since the presence of multiple plasmids is reported to be typical for *Roseobacter* [53, 54]. Gel electrophoresis revealed the presence of mobile elements approximately 50 kb in size, with no smaller elements detected (Supplementary Fig. 1). Gel electrophoresis and hybrid genome scaffolding [55] are suggestive of one chromosome and a second genetic element (Supplementary Fig. 2, Supplementary Table 3). Conversely, in silico plasmid analyses using PlasFlow [38] (mapping-based) and PlasmidFinder [56] (kmer-based) highlighted 8 contigs with different sizes than those inferred from the agarose gel or the hybrid genome scaffolding. To resolve this, a more detailed analysis of the annotations was performed. The high GC content observed in some contigs suggests potential assembly collapses, supporting the hypothesis of fewer, larger replicons rather than numerous smaller ones. In contrast, the scaffolding results suggest the presence of a mobile genetic element that requires further investigation (Supplementary Table 4) [57]. The exclusive presence of virB genes on contigs 5–7 [58], and not on the chromosome, strongly supports their classification as plasmids. Notably, this can explain why database-driven classifiers failed to detect them. Contigs 2, 3, and 4 lack genes for plasmid classification.

### Phylogenetic and phylogenomic analyses

**Fig. 2:**
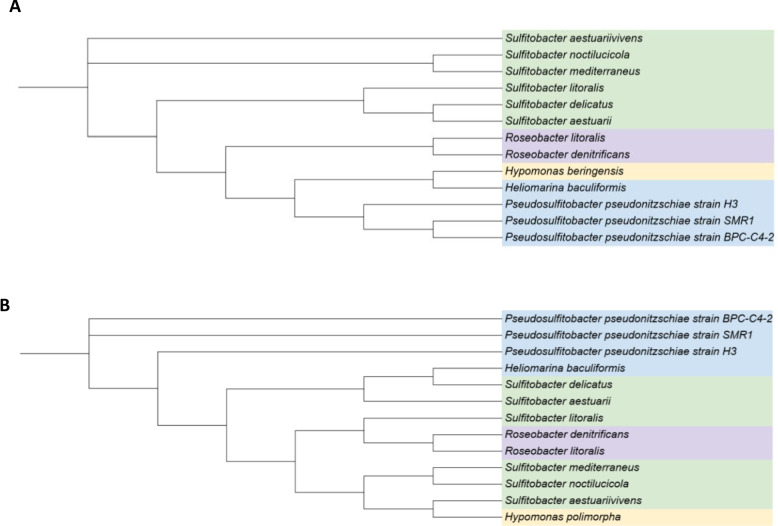
Phylogenetic analysis. **A** Maximum-likelihood phylogenetic tree based on 16S rRNA gene sequences. **B** Phylogenomic tree derived from a distance matrix constructed from the ANI of validated species from the genus *Sulfitobacter* and closely related taxa with available genome sequences. *Hyphomonas* spp. served as the outgroup. Bootstrap percentages from 100 replicates are shown at the nodes, with bootstrap values < 70% indicating low reliability

To assess the effectiveness of the 16S rRNA gene sequence‑based phylogenetic classification of *P. pseudonitzschiae* BPC‑C4‑2 within the taxonomy of *Sulfitobacter*, a maximum‑likelihood phylogenetic tree was reconstructed using 16S rRNA sequences from type strains of the genus *Sulfitobacter* and closely related genera, following previous studies [21] and BLAST‑based suggestions [18] (Fig. 2A).

In this maximum-likelihood phylogenetic tree, the genus *Sulfitobacter* appears paraphyletic due to the inclusion of type strains from the genus *Roseobacter*. Additionally, some branches show insufficient bootstrap support (< 70), indicating low phylogenetic reliability, as reported in previous studies [34]. This suggests that 16S rRNA gene sequences lack the resolution required for accurate phylogenetic reconstruction within *Sulfitobacter* species, thus reinforcing the limitations of single-gene phylogenies in resolving closely related taxa [21].

We generated a phylogenomic tree based on a distance matrix derived from ANI analysis of whole-genome sequences (Fig. 2B). APC identified four distinct genomic clusters, which were visually differentiated by color. APC enabled the unsupervised grouping of closely related bacterial genomes based on genomic similarity, offering a rapid and mathematically sound alternative to traditional clustering methods. The resulting clusters aligned well with annotation-based phylogenetic analyses. Phylogenomic analysis based on ANI supports the classification of *P. pseudonitzschiae* BPC-C4-2 within the genus *Pseudosulfitobacter*. The trees further demonstrate that *Pseudosulfitobacter* forms a clearly defined lineage, distinct from members of the genera *Roseobacter* and *Sulfitobacter*, thus reinforcing the taxonomic separation and clarity among these groups.

Following the phylogenetic classification of growth-inducing BPC-C4-2 within the Pseudosulfitobacter clade, this study investigated the sulfur-oxidation and DMSP-degradation pathways in *P. pseudonitzschiae* BPC-C4-2. Genomic and functional analyses reveal components of the Sox/Soe system and the selective presence of DMSP-related genes, offering insights into the bacterial metabolic potential and ecological role of this strain in sulfur cycling and in adaptation to harsh environments during mutualistic interactions with *Ulva.*

### Genetic analysis linking sulfur cycling and symbiosis

Sulfur serves a central role in global biogeochemical cycles [16]. In the marine electron transport chain, sulfur exists in various oxidation states, ranging from − 2 to + 6, forming compounds such as thiosulfate (S₂O₃^2^⁻), sulfite (SO₃^2^⁻), sulfide (S₂^2^⁻), and sulfate (SO₄^2^⁻). Thus, it enables diverse redox reactions that drive microbial metabolism and energy flow in marine ecosystems. Sulfur compounds, such as DMSP, which is produced by algae as osmolytes, contribute to the marine food web, mediate microbial interactions, and influence atmospheric chemistry by releasing climate-relevant gases such as dimethyl sulfide (DMS) [16]. For example, during green tides, massive *Ulva* blooms release high amounts of DMSP, which *Ulva* itself or the associated bacteria can convert into DMS [59–61]. This microbial transformation significantly contributes to coastal DMS emissions, which influence local sulfur cycling and may impact atmospheric processes such as cloud formation. *P. pseudonitzschiae* BPC-C4-2 is part of this bacterial algae community and is involved in sulfur oxidation, utilizing diverse enzymes and proteins.

*Sulfitobacter* species have been reported to oxidize reduced sulfur compounds [34, 62]. Sulfur-oxidizing bacteria oxidize reduced sulfur compounds to sulfur or sulfate, with the central sulfur oxidation (*SoX*) system capable of oxidizing thiosulfate, sulfide, sulfite, and elemental sulfur to sulfate. We also expected this in *P. pseudonitzschiae* BPC-C4-2. Notably, confusion between two gene systems could lead to significant misinterpretation: SoxXYZABCD is a metabolic pathway for the oxidation of sulfur to obtain energy, whereas SoxRS functions as a protective system against oxidative stress. Instead of SoxXYZABCD, we identified a modular system comprising enzymes for DMSP degradation, together with the *soe* system. However, the ability of *P. pseudonitzschiae* to utilize DMSP as a carbon source requires further investigation, although *Roseovarius* sp. MS2 is not able to use DMSP as a carbon source. Sulfide oxidation also involves enzymes such as flavocytochrome c sulfide dehydrogenase (FCC) and sulfide:quinone reductase (SQR) [63, 64]. These enzymes involved in sulfur oxidation were analyzed by annotation and through KEGG pathway analyses.

Two DMSP utilization routes were identified in the *Pseudosulfitobacter* genome. In the demethylation branch, DMSP is converted to methylmercaptopropionate (MMPA), which is then converted to methanethiol (CH₃SH). The presence of *dmdB*, *dmdC*, and d*mdD* genes supports this pathway [65], while the *mtoX* gene encodes methanethiol oxidase, which converts CH₃SH to formaldehyde and sulfite. The cleavage branch is mediated by *dddL*, producing DMS and acrylate. A conserved FAD-binding oxidoreductase, which is similar to Tmm-like enzymes and consistent with the functions described in related members of the *Roseobacter* clade, likely catalyzes the oxidation of DMS to DMSO [66–69]. This can subsequently yield methanethiol via unresolved intermediates. Notably, both pathways converge on sulfite as a central intermediate.

Sulfite from these processes is oxidized to sulfate by the Soe system, which is encoded by *soeA*, *soeB*, and *soeC*. All parts of the system could be identified via KEGG and Prokka. Under oxic conditions, this cytoplasmic sulfite acceptor oxidoreductase channels electrons into the quinone pool, coupling sulfite oxidation to energy conservation [64].

In the genome, betaine utilization is supported by *betA* (choline dehydrogenase) and betaine transporters (BetT/OpuD-type), which were previously identified in the closely related *Sulfitobacter* genus [70, 71]. Betaine undergoes stepwise demethylation to yield dimethylglycine, sarcosine, and ultimately glycine. Sarcosine is further metabolized into glycine, formaldehyde, and hydrogen peroxide (H₂O₂) via the sarcosine oxidase pathway (*soxA*, *soxB*, *soxD*, *soxG*, and *soxR*), which was identified using Prokka [72]. In *P. pseudonitzschiae*, glycerin may function as an alternative carbon and energy source during extended winter darkness, thereby supporting growth and metabolic activity, potentially in association with sulfate reduction (Table 2) [73].

**Table 2:** Annotation and functional analysis of key sulfur metabolism genes (*dmd*, *sox*, *soe*), and ectoine (*ect*) metabolism genes, methylation, and demethylation, including results from Prokka annotation, sequence alignment, and KEGG pathway identification

	**Functional Annotation (Prokka)**	**Pathway Analysis (KEGG)**
*dmdA*	**-**	**-**
*dmdB*	** + **	**-**
*dmdC*	** + **	**-**
*dmdD*	** + **	**-**
*dddL*	** + **	**-**
*dddP*	**-**	**-**
*acuK*	**-**	**-**
*motX*	** + **	**-**
*dddA*	**-**	**-**
*dddC*	**-**	**-**
*soxA*	** + **	**-**
*soxB*	** + **	**-**
*soxD*	** + **	**-**
*soxG*	** + **	**-**
*soxR*	** + **	**-**
*soeA*	** + **	** + **
*soeB*	** + **	** + **
*soeC*	** + **	** + **
*ectA*	-	**-**
*ectB*	-	**-**
*ectC*	-	**-**

Remarks highlight multiple annotations, regulatory relationships (e.g., AcuK/AcuC), and potential replacements within the Sox system by the Soe system. + indicates the presence of the gene, while—indicates its absence

The comparison of KEGG and Prokka annotations underscores differences in pathway reconstruction: Prokka provides gene-centered functional assignments, whereas KEGG emphasizes pathway-level integration. When combined, these approaches offer complementary insights into the metabolic potential of the organism. The results were compared with the *sox* gene cluster of *Sulfitobacter pseudonitzschiae* strain SMR1. In both genomes, *soxA*, *soxB*, *soxD*, *soxG*, and *soxR* were identified, predominantly located within the putative chromosomal region of our assembly, except for one copy of soxA and one copy of soxB on contig 4. Interestingly, the genes are in the same order in both strains, indicating evolutionary stability. Notably, SMR1 displayed a comparable arrangement of *soxA* and *soxB* on a contig of identical size. The genes *soxS* and *soxH* were only annotated in SMR1 by Prokka and KEGG. However, mapping *soxH* against our assembly revealed a 100% match, annotated as a “putative gene” in our genome. Furthermore, *soxS* mapped with 100% identity but corresponded to the homologue *rhaS*, which, based on its position and size, was classified as *rhaS* rather than *soxS*, despite the high similarity of their receptor families.

Although sulfur oxidation and DMSP catabolism are central metabolic features of SMR1 and likely important in its association with the host, comparative genomics suggest that *Pseudosulfitobacter* strains do not rely on the same interaction mechanism. For example, the strain of *P. koreense* associated with the alga *Alexandrium* contains a chromosomally encoded sxt gene cluster that is thought to contribute to saxitoxin biosynthesis [21]. SMR1 possesses a complete indole-3-acetic acid (IAA) pathway reported to promote diatom growth via auxin [49]. However, the *Ulva-*associated *Pseudosulfitobacter* analyzed here lacks both the sxt gene cluster and the canonical IAA biosynthesis genes. *Pseudosulfitobacter* interaction traits diverge across hosts, with different signaling and metabolic functions potentially being distributed among co-residing bacteria within each algal holobiont.

### Comparative genome alignment and structural variation analysis

To elucidate the genomic structure of the genes and mobile elements, we performed a comparative genome alignment and structural variation analysis. *P. pseudonitzschiae* BPC-C4-2 and other analyzed members of the genus exhibit high GC content and a large number of plasmid-like structures. These factors are known to challenge assembly algorithms, often resulting in structural misassemblies or incomplete contig resolution [74]. To mitigate these risks and evaluate the influence of assembly strategy on genome completeness and structural accuracy, two additional assemblers were applied alongside our initial Flye assembly for direct comparison: Unicycler and Canu.

Annotation with Prokka revealed that the Unicycler and Flye assemblies exhibited very similar predicted genomic content. Unicycler assemblies contained 5,373 CDS, six rRNA genes, 85 tRNA genes, and one tmRNA gene. The Flye assembly differed only slightly, containing 5,380 CDS and sharing the same rRNA, tRNA, and tmRNA counts as Unicycler. In contrast, the Canu assembly contained a notably higher number of predicted features, including 5,679 CDS, nine rRNA genes, 89 tRNA genes, and one tmRNA gene (Supplementary Table 5). This elevated number of gene predictions for Canu is consistent with its larger total genome size and may indicate the presence of additional genomic content, such as plasmid-derived sequences, or the introduction of duplication artifacts during assembly.

All seven contigs resulting from the Flye and Unicycler assemblies could be circularized. This suggests that the contigs are plasmid-like, as proposed in other publications, even though PlasmidFinder and PlasFlow were unable to identify them as plasmids. Notably, the Canu assembly did not result in any circular structures.

**Fig. 3:**
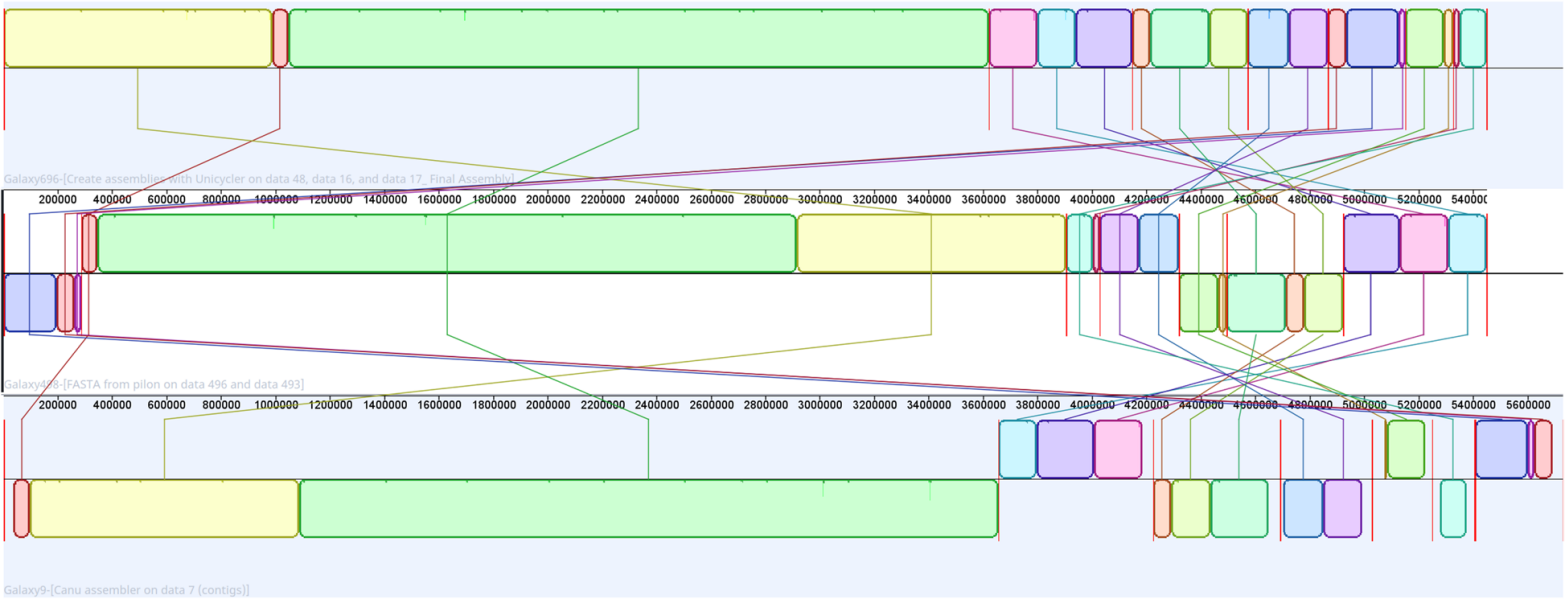
Schematic representations of *P. pseudonitzschiae* sequence alignment. Each contiguously colored region represents a colinear block (i.e., a region without rearrangement of the homologous backbone sequence). Lines between genomes trace each orthologous colinear block across all genomes. Colinear blocks below a genome's center line represent segments that are inverted relative to the reference genome

Whole-genome alignment of the three assemblies in Mauve identified 35 locally collinear blocks (LCBs) shared by all datasets. Rearrangement distance analysis based on shared LCBs further clarified these relationships. Canu was structurally closer to Unicycler (double cut and join (DCJ) distance = 13) than to Flye (DCJ distance = 20), while the distance between Unicycler and Flye was moderate (DCJ distance = 19). Single-Cut-or-Join distance (SCJ) were uniformly high (70) for all comparisons, and breakpoint distances were identical (35) for each pairwise comparison. This indicates that, although the number of disrupted adjacencies is consistent across assemblies, the complexity and type of rearrangements differ (Supplementary Table 6, Fig. 3).

Overall, these results demonstrate that although Unicycler and Flye produce nearly identical genome sizes and gene content, the Canu assembly captures more sequence and predicts more features, albeit at the expense of introducing additional rearrangements. Depending on the data collected, Flye or Unicycler assemblies should be used for downstream analysis based on data completeness and circularization.

Current models of *Ulva* morphogenesis have mainly been established in temperate systems and focus on the identity and combination of bacterial algal growth- and morphogenesis-promoting factors, such as thallusin and cytokinin-like compounds [48]. While these studies demonstrated that *Ulva* development fundamentally depends on bacterial partners, they largely neglect environmental constraints and therefore provide limited explanatory power for polar regions, where low temperatures restrict bacterial growth, enzymatic activity, and metabolite production [6].

Our results extend these models by placing *Ulva* morphogenesis in a polar context and linking developmental outcomes to the genomic and metabolic capacities of cold-adapted bacterial symbionts. The induction of *Ulva* morphogenesis by *Pseudosulfitobacter pseudonitzschiae* BPC-C4-2, in combination with Maribacter, indicates that morphogenetic functionality can be maintained under cold conditions, provided that the associated bacteria remain metabolically active [2].

Genomic analyses revealed metabolic traits related to sulfur compound turnover, DMSP degradation, and osmolyte metabolism, which likely support bacterial persistence and activity in cold, chemically dynamic environments. Together, these findings support a shift from a purely morphogen-centered view towards an environment-specific holobiont perspective, in which *Ulva* morphogenesis in polar regions emerges as an integrated property of a cold-adapted algal–bacterial consortium.

### Limitations of the study

While this study provides new insights into the genomic architecture and metabolic potential of *Pseudosulfitobacter pseudonitzschiae* BPC-C4-2, several limitations should be acknowledged. The reconstruction of replicon structure in plasmid-rich, high-GC marine bacteria remains challenging, and the combined evidence from electrophoretic profiling, in silico prediction tools, and long-read assemblies does not always converge on a single, unambiguous interpretation. While the enrichment of conjugation-associated genes (e.g., *virB*) is consistent with plasmid-associated mobility, such markers alone are not definitive. Accordingly, plasmid assignments presented here should be considered likely rather than definitive, and future improvements in long-read sequencing accuracy and assembly algorithms will be essential to fully resolve replicon organization.

Similarly, the interpretation of mobile genetic elements and plasmid-associated functions is constrained by the inherent limitations of current computational classifiers and by the modular nature of plasmid gene content. While markers such as *virB* provide useful indications of conjugative potential, they do not by themselves define plasmid identity, and our conclusions therefore emphasize probabilistic rather than categorical assignments.

With respect to the integration of microbial genomics and algal biology, this work focuses on the bacterial genomic traits that enable morphogenetic interactions under polar conditions. Although direct host transcriptomic responses were not assessed, the genomic and metabolic features identified here provide a mechanistic framework for understanding how bacterial partners can sustain *Ulva* morphogenesis under cold stress. We thus provide a foundation for future experimental work that directly links bacterial genomic potential to host developmental regulation in polar marine holobionts. The putative metabolic roles inferred from gene content and comparative annotation must be validated by future physiological or expression-based assays.

## Conclusions

This study establishes a tripartite system to explore the morphogenetic influence of cold-adapted bacteria (i.e., *Maribacter* and *Pseudosulfitobacter*) on *Ulva mutabilis*. Phylogenetic analyses identify the closest genetic relatives of these strains as taxa occurring in the Baltic Sea and the Korean Sea. Affinity propagation clustering enabled a more robust phylogenetic placement of *P. pseudonitzschiae* within this phylogenetically challenging group. Moreover, gene and tRNA content results indicate broad adaptations to cold environments. Although *Pseudosulfitobacter* species primarily use the sox gene clusters for sarcosine oxidation rather than classical sulfur oxidation, their genomes also encode multiple pathways involved in the turnover of reduced sulfur compounds. Together, these features enhance our understanding of the metabolic flexibility of *Pseudosulfitobacter* species and their potential role in marine sulfur cycling, highlighting their broader significance beyond morphogenesis in *Ulva*.

## Supplementary Information

Supplementary Material 1.

## Data Availability

The datasets generated and/or analyzed during the current study are available in the BPC-C4-2 repository and through the following NCBI links: https://www.ncbi.nlm.nih.gov/bioproject/PRJNA828511; https://www.ncbi.nlm.nih.gov/biosample/SAMN47449418; https://www.ncbi.nlm.nih.gov/nuccore/JBMGNR000000000; and https://www.ncbi.nlm.nih.gov/Traces/wgs/JBMGNR01?display=contigs.

## Abbreviations

AGMPF: Algal growth and morphogenesis-promoting factors
ANI: Average nucleotide identity
APC: Affinity propagation clustering
CDS: Coding sequences
DCJ: Double cut and joi
DMSP: Dimethyl sulfoniopropionate: DMSP
DMSOP: Dimethylsulfoxoniumpropionate: DMSOP
DMS: Dimethylsulfide
DMSO: Dimethylsulfoxide
LCB: Locally collinear block
LPSN: List of prokaryotic names with standing in nomenclature
MMPA: Methylmercaptopropionate
mtRNA: Mitochondrial ribonucleic acid
ONT: Oxford Nanopore Technologies
SCJ: Single-Cut-or-Join distance
soe: Sulfite-oxidizing enzyme
sox: Sarcosine oxidase
tRNA: Transfer ribonucleic acid
rRNA: Ribosomal ribonucleic acid
