# Supplementary material for: Genomic and functional characterization of Pseudosulfitobacter pseudonitzschiae BPC-C4-2: a growth-promoting symbiont in Antarctic Ulva communities
Source: BMC Genomics. 2026 Feb 7;27:230. doi: 10.1186/s12864-026-12626-w (PMC12930554; doi:10.1186/s12864-026-12626-w)
Supplement: Supplementary file 1 — Supplementary Material 1. [file 12864_2026_12626_MOESM1_ESM.pdf]

Supplemental Figures and Tables

Antarctic *Pseudosulfitobacter pseudonitzschiae* BPC-C4-2: Genetic Analysis and Application in a Cold-Water Morphogenic *Ulva* System

Tia Wünschmann[1,2], Fatemeh Ghaderiardakani[3], Timo Homeier-Bachmann[4], Maria Lilliana Quartino[5], Thomas Wichard[3*], Anne Busch[1,2,*]

[1] Theoretical Microbial Ecology, Friedrich Schiller University, Jena, Germany

[2] Cluster of Excellence Balance of the Microverse, Friedrich Schiller University Jena, Jena, Germany

[3] Institute for Inorganic and Analytical Chemistry, Friedrich Schiller University, Jena, Germany

[4] Friedrich-Loeffler-Institute, Federal Research Institute for Animal Health, Institute of Epidemiology, Friedrich-Loeffler-Institute, Greifswald-Insel Riems, Germany.

[5] Department of Coastal Biology, Argentinean Antarctic Institute, Buenos Aires, Argentina.

*Corresponding authors: thomas.wichard@uni-jena.de and anne.busch@uni-jena.de

Supplemental Table 1:

Strain	Acession Number 16S	Acession Number WGS
Sulfitobacter aestuariivivens	NR_181018	NZ_JACTAG010000000
Sulfitobacter noctilucicola	NR_134206	NZ_JASD01000001
Sulfitobacter mediterraneus	NR_026472	NZ_OMPT01000000
Sulfitobacter litoralis	NR_043547	NZ_JAXMOT010000000
Sulfitobacter delicatus	NR_025692	NZ_FNBP01000000
Sulfitobacter aestuarii	NR_179825	NZ_JBHUMP010000000
Roseobacter litoralis	NR_074143	NZ_CANLWU010000000
Roseobacter denitrificans	NR_102909	NZ_FOOO01000000
Heliomarina baculiformis	NR_181734	NZ_JAIMIC010000000
Hypomonas beringensis	/	NZ_AWFF01000000
Hypomonas polimorpha	AJ227813	/
Pseudosulfitobacter pseudonitzschiae strain H3	NR_178596	NZ_JAMD01000000

Pseudosulfitobacter pseudonitzschiae strain SMR1	Blast from WGS	CP022422

Supplemental Table 1: Name and Accession Numbers of strains used in comparative genomic analysis.

Supplemental Table 2:

	ONT/Illumina hybrid assembly	Illumina assembly
contigs	7	76
largest contig	3619192	758745
total length	5450390	5404620
N50	3619192	203213
N90	284771	56481
auN	2526817	302086
L50	1	8
L90	50	25
GC	58,98	58,99

Supplemental Table 2: The table compares hybrid and Illumina assemblies, highlighting differences in contig count, total bases, scaffold sizes, GC content, and assembly metrics such as N50 and gaps, demonstrating the improved contiguity and accuracy of the hybrid assembly

Supplementary Figure 1

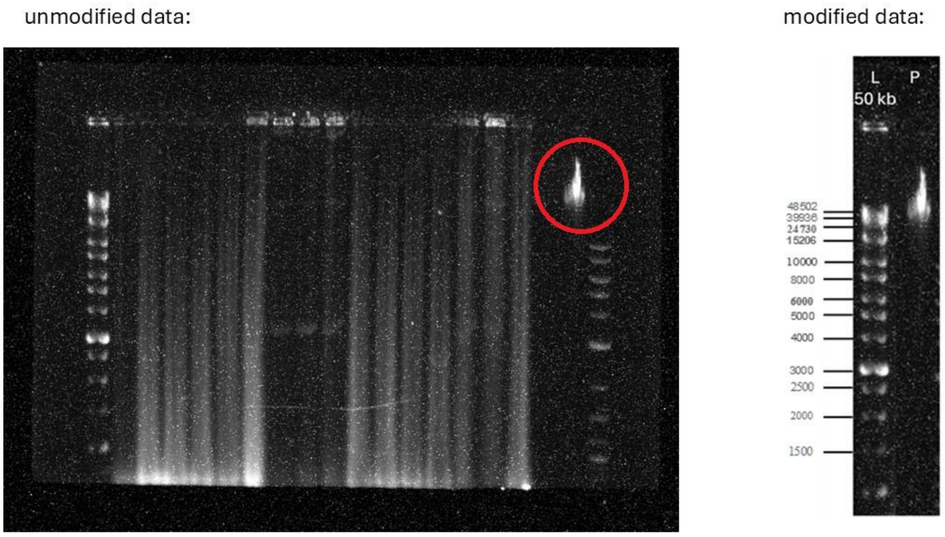

Supplemental Figure 1: Plasmid Preparation of *P. pseudonitzschiae* BPC-C4-2. Plasmid DNA extracted from *P. pseudonitzschiae* BPC-C4-2 was resolved on a 0.5% agarose gel and visualized by staining with ethidium bromide. A 1 kb ladder was run in parallel to serve as a molecular weight reference, highlighting the estimated sizes of the plasmid bands.

Supplementary Figure 2:

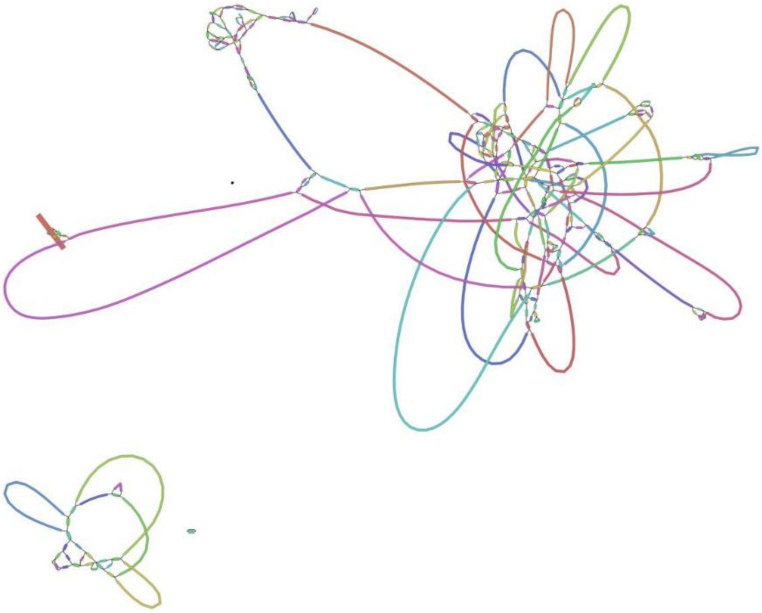

Supplemental Figure 2: Bandage visualization of the hybrid genome assembly. A graphical representation of assembly graphs, enabling to explore contig connections, detect possible misassemblies, and assess overall assembly quality.

Supplementary Table 3:

Pseudosulfitobacter	contig 1	contig 2	contig 3	contig 4	contig 5	contig 6	contig 7
length (bp)	425393	292931	175267	284771	528791	3619192	124045
PlasmidFinder	unclassified	unclassified	unclassified	unclassified	unclassified	unclassified	unclassified
Plasflow	unclassified	Chromosome	Plasmid	Plasmid	unclassified	Chromosome	Plasmid

Supplementary Table 3: Summary of mobile element analysis including contig length (bp) and *in silico* plasmid predictions using PlasmidFinder and PlasFlow.

Supplementary Table 4:

Contig	GC Contig in %
1	58.3
2	58.8
3	59.7
4	58.4
5	53.7
6	59.9
7	53.7

Supplementary Table 4: Summary of GC contents in each contig in %.

Supplementary Table 5:

Assembly	Contigs	Genome Size (bp)	CDS	rRNA	tRNA	tmRNA	Circularisation
Unicycler	7	5,450,319	5,373	6	85	1	All contigs
Flye	7	5,450,390	5,380	6	85	1	All contigs
Canu	9	5,774,190	5,679	9	89	1	none

Supplementary Table 5: Comparison of Pseudosulfitobacter pseudonitzschiae BPC-C4-2 assembled by Unicycler, Flye and Canu across contig number, differences in base pairs, CDS, rRNA, tRNA, tmRNA, GC content and Circularisation.

Supplementary Table 6:

Assembly Comparison	DCJ Distance	SCJ Distance	Breakpoint Distance
Unicycler – Flye	19	70	35
Unicycler – Canu	13	70	35
Flye – Canu	20	70	35

Supplementary Table 6: Rearrangement distances between the three assemblies based on 35 shared LCBs.
